# The Effect of Motor Imaginary Combined with Transcranial Direct Current Stimulation (tDCS) on Balance in Middle-Aged Women with High Fall Risk: A Double-Blind Randomized Controlled Trial

**DOI:** 10.1155/2023/9680371

**Published:** 2023-03-31

**Authors:** Esmaeil Mozafaripour, Seyed Kazem Mousavi Sadati, Leila Najafi, Maryam Zoghi

**Affiliations:** ^1^Department of Health and Sports Medicine, Faculty of Physical Education and Sport Sciences, University of Tehran, Tehran, Iran; ^2^Department of Physical Education and Sport Science, East Tehran Branch, Islamic Azad University, Tehran, Iran; ^3^Discipline of Physiotherapy, Institute of Health and Wellbeing, Federation University Australia, Victoria, Australia

## Abstract

**Introduction:**

The risk of falling and its subsequent injuries increases with aging. Impaired balance and gait are important contributing factors to the increased risk of falling. A wide range of methods was examined to improve balance, but these interventions might produce small effects or be inapplicable for this population. The current study aimed at investigating the effect of motor imaginary (MI) training combined with transcranial direct current stimulation (tDCS) over the cerebellum on balance in middle-aged women with high fall risk.

**Methods:**

Thirty subjects aged 40-65 years old were divided into two groups including intervention (*n* = 15) and sham control (*n* = 15). The participants completed a 4-week program 3 times per week. The intervention group performed MI training combined with tDCS over the cerebellum, and the control group performed MI training combined with sham tDCS over the cerebellum. Static and dynamic balance were measured at baseline and after completing the 4-week program using balance error scoring system (BESS) and Y balance testing, respectively.

**Result:**

A one-way analysis of covariance and paired *t*-tests were used to analyze the data. Significant improvement was observed in both balance tests in the intervention group after the implementation of the 4-week intervention program compared to the control group. The within-group analysis showed that both static and dynamic balance improved significantly from the baseline values only in the intervention group (*p* < 0.05) and not in the control group (*p* > 0.05).

**Conclusion:**

The results of the study indicate that MI training combined with tDCS over the cerebellum can lead to balance improvement in middle-aged women with high fall risk.

## 1. Introduction

Falls are a serious health-related problem among men and women. It has been estimated that almost one-third of the older adult population has experienced at least one fall per year, and injuries from falling are more likely to occur as you age [1]. Fall is considered one of the most important causes of hospitalization in older adults [2].

The consequence of a fall in this population can be dangerous. In many cases, falling leads to severe limitations in daily activities and loss of independence in older adults, as well as implies an excessive burden on their families and the health care system [2]. Even though most of the previous studies have evaluated the risk of falling in elderly individuals, recent studies have shown that the risk of falling is increasing in middle-aged adults too [3]. Moreover, the previous studies revealed that women as compared to men have a higher rate of nonfatal falls and fall-related injuries in all age groups [1, 4].

On the other hand, osteoporosis and decreased bone health are inevitable consequences of aging in women due to menopause [5]. In previous studies, impaired balance and gait were identified to be the most important risk factors for falls [6]. Additionally, entering to menopause status is associated with decreased muscle strength and changes in body composition and body fat distribution. All of these factors contribute to decreased balance and increased fall risk in middle-aged women [7, 8]. As they age, the risk of falling will increase even more. Therefore, it is evident from all of the factors discussed above that preventing falls among women in this age group is important. Hence, any intervention, which improves balance and postural control in this population, may play an important role in the reduction of falls and fall-related injuries.

A wide range of research has examined the effects of several methods such as specific balance training and strength and functional training on balance improvement. The results showed that these kinds of interventions might produce small effects and benefits or be inapplicable in older adults [9, 10]. The use of less physically demanding methods and interventions has increased over the past few years [11, 12], such as motor imaginary (MI) training, which is much easier to use in making prevention programs more effective. This has the potential to improve balance and mobility-related factors in adult population, including middle-aged women. MI is a cognitive state that concerns the process of imagining a motion without its physical executions [11]. MI elicits activity in brain areas that are generally activated through real task mission performance [12]. It has been shown that MI can be beneficial for older people because of its abilities to promote motor learning and activity-dependent plasticity [13].

It may be necessary to add MI training to the physical training program so they are able to complete enough repetitions to have a beneficial effect since they may not be able to follow the recommended physical routine. However, the result of a recent review on this topic revealed that although these methods can improve balance in older adults, the effect size of these protocols is not clinically worthwhile [14].

Furthermore, noninvasive brain stimulation has demonstrated effectiveness in improving balance and posture [15, 16]. Transcranial direct current stimulation (tDCS) is a noninvasive neuromodulatory intervention which can be easily implemented [16]. In a review study, Baharlouei et al. reported that tDCS intervention may improve dynamic balance in young healthy individuals and showed a positive effect on older adult's balance. However, there is no definitive consensus. They reported that while there is a lack of evidence to support the effectiveness of tDCS interventions on static balance task in older adults in single-task conditions, it seems that applying tDCS on dorsolateralprefrontal cortex (DLPFC) could improve static balance in dual-task conditions in older adults. They concluded at the end that due to the limited published studies in this area and many controversies on the findings of these studies, caution should be utilized when drawing any conclusion on applying these methods [17]. Therefore, we hypothesized that combining the MI training with tDCS intervention on the cerebellum (which is the most important area in balance control) could have cumulative effects on balance indexes in middle middle-aged women.

The aim of this study is to investigate the effectiveness of MI training combined with tDCS over the cerebellum on improving balance in middle-aged women.

## 2. Materials and Methods

### 2.1. Study Design

The present study was a double-blind randomized controlled trial. It was conducted in accordance with the ethical standards of the Helsinki Declaration. The study's ethical approval was obtained by the Ethics Committee on Research at the Sport Sciences Research Institute, Tehran, Iran. Sample size was estimated through G^∗^Power software (Version 3.1.9.2; Kiel, Germany). According to the previous similar studies, the researchers concluded that in each group, 15 participants were needed to detect the statistical significance at the level of 0.05 and a statistical power of 80% [18, 19]. In this study, an informed consent form was signed by each participant before starting the trial.

### 2.2. Participants

A total of 75 volunteers were informed about the study through advertisements and screened against inclusion/exclusion criteria to enroll in the study. The inclusion criteria included middle-aged adults aged 40-65 years who scored five or higher on the basis of the fall risk assessment scale [20] and being at least one SD above the mean score of the MI questionnaire of all subjects who screened for eligibility [21]. The exclusion criteria included having any history of neurological diseases or psychological illnesses; the use of any medications that can affect their balance control such as sedative medications; the presence of any lower extremity injuries such as fracture, ligament injury, muscle strain, or low back pain that restrict movements [22, 23]; the presence of any sign of spinal cord involvement; any visual or auditory impairments; any musculoskeletal deformities in lower or upper extremities that could affect participant's posture in standing [24, 25]; any skin conditions (e.g., eczema and lesions) on scalp, and the presence of metal inside the head (outside the mouth) such as shrapnel, surgical clips, or fragments from welding or metalwork; any implanted devices such as cardiac pacemaker, cochlear implant, medical pump, or intracardiac line; or losing more than three training sessions or two consecutive sessions.

**Figure 1:**
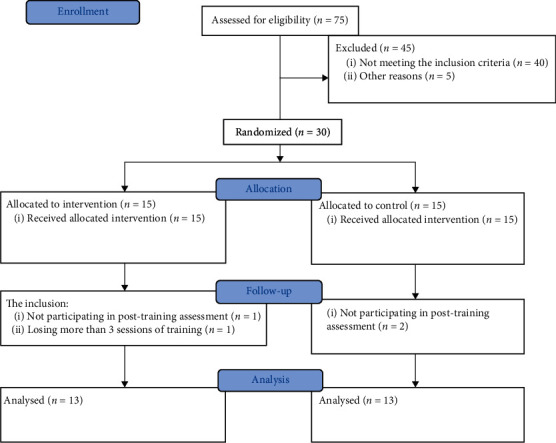
CONSORT flow chart.

Thirty volunteers, as shown in Figure 1, met the inclusion criteria. They were randomly assigned into either an intervention or control group. Participants were selected using computer-generated block randomization in a 1 : 1 ratio. This process was followed by a concealed allocation through opening the sequentially numbered, opaque, and sealed envelopes, and a card inside indicating the group into which the participant was randomly allocated, i.e., the intervention or control group.

### 2.3. Intervention

The intervention started 48 hours after the baseline balance assessments. Each intervention session lasted 30 minutes. At the beginning of each session, both groups were asked to perform MI exercises for 15 min. Then, all participants in both groups received a-tDCS or sham tDCS for 15 min. Balance tests were completed 48 hours before the first intervention session and after the last intervention session. Both researchers and the participants were blinded to the participant's allocation to each group in this study.

**Figure 2:**
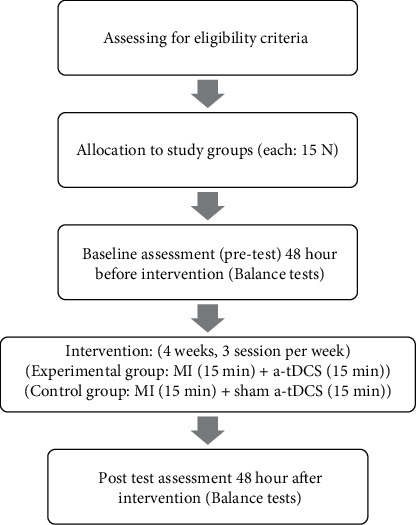
Procedure of the study.

All participants completed a 4-week intervention program (3 times per week). In sum, the intervention consisted of 12 sessions. Every 2 sessions were at least 48 hours apart from each other. Procedure of the study is illustrated in Figure 2.

### 2.4. MI Training

Ability of MI in subjects was assessed by revised version of Movement Imagery Questionnaire (MIQ-R). This questionnaire is composed of three subscales: assessing internal visual imagery, external visual imagery, and kinesthetic imagery. As in the original questionnaire (MIQ-R), the first four basic movement descriptions were read and physically performed by subjects, followed by imaging of the three subscales mentioned above. Then, the subjects rate their MI ease on a 7-point Likert-type scale ranging from 1 (very hard to see/feel) to 7 (very easy to see/feel). An average score for the entire subscales of questionnaire was calculated by the examiners, with higher score representing greater ease of imaging [26]. We include subjects in this study who were at least one SD above the mean of all subjects screened for eligibility.

To perform MI training, cognitive specific imagery method was used [27]. For this purpose, a quiet room was provided for the participants. Before running the MI exercises, they were asked to watch a recorded video featuring balance tests being performed correctly, assuming that they were performing them themselves. Then, they were asked to lie on the bed and close their eyes. In order to provide the participants' concentration, each session started with a relaxation process for 5 min [28], and in the next 10 min, the participant was asked to concentrate on imagining the performance of balance test and try to feel the sensations that occur when performing the tasks [29]. At the end of each session, the examiner verbally asked about the quality of the MI training session from each subject, and if needed, they were given the necessary guidance for a better implementation of the MI intervention in the following session.

### 2.5. Transcranial Direct Current Stimulation (tDCS)

**Figure 3:**
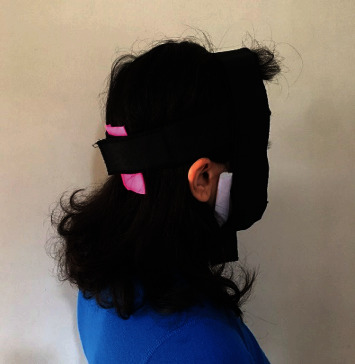
Electrode montages for tDCS. For a-tDCS of the cerebellum, active electrode (anode) was placed 1 cm below inion to target the cerebellum and the returning (cathode) electrode was placed over the right buccinators muscle (taken by authors).

Participants in the intervention and control groups received real tDCS (2 mA) and sham tDCS over the cerebellum, respectively, at each session after the MI training session for 15 minutes. TDCS was administered using a Neurostim2 tDCS device through a pair of rubber electrodes (5 × 7 cm) enclosed in saline-soaked sponge pockets. The active electrode (anode) was placed 1 cm below inion (Iz) to target the cerebellum, and the returning (cathode) electrode was placed over the right buccinator muscle [16]. The electrode placements in the control group were exactly similar to the intervention group; however, participants in this group received the current for 30 s, and it was slowly turned off after that (fade-in short stimulation fade-out approach) [16, 30] (Figure 3).

### 2.6. Balance Assessment

#### 2.6.1. Y Balance Testing

**Figure 4:**
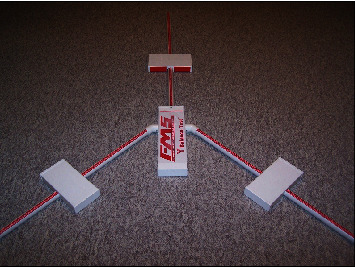
Y balance test kit (taken by authors).

Y balance test was applied for assessing the dynamic balance of participants. This test has been considered as a reliable and valid test for balance assessment in older adults [31]. In order to administer this test, the participants were asked to stand with one leg on the starting block and use their other leg, as a reach leg, to push forward the reach indicator pin as far as possible in the three directions of anterior, posterolateral, and posteromedial. Attempts were not saved if the participants were unable to keep a single-leg stance throughout the whole trial, if they rested the reaching foot on top of the indicator pin while moving it forward, if they kicked the indicator pin forward in an attempt to reach extra distance, or if they could not return to the starting position while maintaining balance [32]. Prior to running the test, the participants warmed up sufficiently for 5 minutes, and they had 3 testing trials to become familiar with how to run the test [31]. Each subject performed 3 trails of the test. The average of these three trails was recorded as each participant's score. Participants were allowed to take a brief rest between each attempt to prevent fatigue. Scores of reaching direction were normalized by dividing the average reaching distance (in cm) of each participant on lower leg length (in cm). Moreover, this score was multiplied by 100 to get the percentage of leg length. The participants' leg length was measured from the anterior–superior iliac spine to the medial malleolus. Finally, to obtain a combined score of the three directions, the sum of the average of three trials in the three directions was divided to three times of the leg length [33] (Figure 4).

#### 2.6.2. Balance Error Scoring System (BESS)

In order to evaluate static balance in participants, BESS test was used. This test comprises 3 standing postures: double leg stance, single leg stance, and tandem stance. These three standing postures are performed with hands on the hips and closed eyes on two different surfaces, namely, firm surface and foam surface. Each participant's score was calculated based on an error system as the number of errors of each participant was recorded as his score. The errors were as follows: lifting hands from the hips, opening the eyes, falling, inability to return to standard position in more than 5 seconds, lifting any part of the foot from the surface, and hip adduction more than 30 degrees [34, 35]. The whole testing process was done by an expert, and test-retest reliability for this assessment was completed in a pilot study (ICC = 0.85).

## 3. Statistical Analysis

All statistical analyses were conducted using SPSS statistical software (Chicago, Illinois, V.22).

A one-way analysis of covariance (ANCOVA), with regard to the pretest measurement as the covariate, was utilized to investigate the differences between the experimental and sham control groups. In addition, the paired *t*-test was used to compare within-group differences in pre- and posttests. Effect size was calculated in partial eta-squared (*η*^2^). Effect sizes were interpreted as small (0.01), medium (0.09), or large (0.14) [36]. The significant difference was set at *p* < 0.05.

## 4. Results

The results of the Shapiro-Wilk test of normality and *t*-test revealed that the demographic variables (age, height, and weight) in this study had normal distribution between the groups at baseline, and there were no significant differences between groups regarding the demographic variables (Table 1). MIQ-R questionnaire global score (M ± SD) for the intervention and control groups was 44.48 ± 2.1 and 40.20 ± 6.2, respectively. Before performing the ANCOVA analysis, the assumption of homogeneity of regression slopes was tested. The results indicated that the interaction between intervention and pretest is not significant for either variable (BESS test (score): *F* = .64, *P* = .72 and Y test (cm): *F* = 1.5, *P* = 0.23).

Descriptive statistics of the studied variables, the results of the one-way ANCOVA analysis, and the results of paired *t*-test are provided in Tables 2–4, respectively. The results of the analysis of covariance test adjusted for baseline characteristics showed a significant difference between the research variables (*p* < .05). In addition, the results of both tests indicated a significant improvement in the intervention group compared to the sham control group in the postintervention test.

The results of paired *t*-test for the BESS test and Y test score in the intervention group showed that there was a significant difference between before and after the intervention (*t* = 3.88, *p* = .002 and *t* = 2.85, *p* = .04, respectively). This indicates that the balance indexes of participants with balance deficiency were significantly enhanced after receiving interventions.

## 5. Discussion

The findings of the present study confirmed the hypothesis, indicating that, compared to sham tDCS, the MI training combined with tDCS intervention over the cerebellum significantly improved the balance indices of middle-aged women with high fall risk. The findings also showed that the balance indexes did not improve after MI with a sham tDCS compared to baseline.

In this study, participants in the control group received sham tDCS in addition to MI training for 4 weeks. As the assessed balance indexes did not show any significant changes, actually obtained data indicates that MI alone, at a group level, had no significant effect on the assessed balance indexes. The result of the current study is consistent with what Linden et al. and Batson et al. showed in their studies. They concluded that mental practice alone has little or no effect on balance in older adults [37, 38] They point to some methodological issues in explaining the obtained results such as the inability to keep the subjects interested during the intervention, lack of control prior knowledge of mental practice, subjects' mood, personal distractions, sickness, level of hunger or fatigue, and/or response to weather. All these factors can be applied to this study as well. On the other hand, Chiacchiero et al., Goudarzian et al., and Hamel and Lajoie reported that MI training had positive and statistically significant effect on balance indexes in the elderly population [39–41]. In addition, Nicholson et al. in a systematic review reviewed 12 randomized control studies including 356 participants that all investigated the effects of MI training on balance indexes in older adults. They reported that despite the fact that MI training could improve balance indexes in older individuals, because of study design limitations in reviewed studies such as low sample sizes, nonblinded assessors, nonconcealed allocation, and not declaring effect sizes, it is not clear whether these improvements are significant enough to warrant clinical consideration [14].

The neuromuscular theories of MI [42, 43] suggest that the mental imagining of a task results in weak neuromuscular activities in all the muscles that are active when the task is actually performed. These weak neuromuscular activities can eventually lead to the improvement of motor performance during the actual task [44]. Thus, MI training facilitates the function of the neural pathways, which are involved in performing the movement in the real conditions, and therefore, it has been suggested that it would improve the muscle functions during the actual movements. However, tailored MI training protocols need to be developed and assessed for different age groups in future studies.

### 5.1. Application of tDCS on the Cerebellum

Cerebellum has an important role in postural control [45, 46]. In this region, the sensorimotor information, including visual, vestibular, and proprioceptive inputs, is integrated and used for postural control and controlling coordinated movements [17, 45]. In addition, there is a functional connectivity between the cerebellum and motor cortex which has a noticeable effect on cognitive and motor task performances [47]. It has been shown that aging has a negative impact on some areas in the cerebellum, e.g., vermis area, which is responsible for postural control [48] and overall cerebellar functions [49, 50].

In this study, we applied the tDCS on the cerebellum on 12 sessions over a 4-week period with a significant effect on balance indexes. In line with previous studies such as Inukai et al., Hupfeld et al., and Kaminski et al., the findings of the current study confirm that applying tDCS can improve balance indexes [18, 51, 52].

It has been shown that applying tDCS on the cerebellum enhances functional connectivity between the cerebellum and motor cortex, which can finally lead to improving the integration of information and postural control and developing the quality of connection between the cerebellum and other parts of the brain [16, 30, 51]. Furthermore, it had a positive impact on volume and function of the vermis area [48, 53, 54] which could have a positive effect on postural control and balance indexes.

Additionally, the present study revealed that the combination of MI training and applying tDCS over the cerebellum had significant effects on improving balance indexes. There is a possibility that the effect of this combined intervention on balance indexes was just due to the effect of tDCS over the cerebellum only, and MI training did not have any effect on these indexes at all. However, we need to consider that as this intervention was delivered 3 times per week for 4 weeks, the tDCS sessions might act as a priming intervention for the following MI training session especially when they gradually develop some accumulative effects and therefore increase the MI training effect on these balance indexes. This possibility needs to be assessed in future studies as we did not have a group of participants who just received tDCS over the cerebellum to be able to compare the results between the groups.

## 6. Limitations of the Study

We did not assess MI training or tDCS over the cerebellum alone in this study, so it is impossible to comment on the effect of these interventions individually or in combination in middle-aged women with high fall risk. Although we examine the success or quality of MI training verbally at the end of each session, we have not used an instrument to assess quantitatively the success or quality of MI similar to another study in the literature [29], so it is not possible to definitively conclude that the MI training in participants was performed with high quality, and this issue can overshadow the obtained result. Current study sample was consisted of 15 participants in each group, and maybe larger sample size would lead to different results.

## 7. Conclusion

The findings in the current study indicate that MI training combined with tDCS over the cerebellum interventions for 4 weeks can improve static and dynamic balance in middle-aged women with high fall risk. Clinicians and practitioners may consider MI training combined with tDCS as a less physically demanding method for improving balance and reducing fall risk in older adults. Moreover, the results also indicate that 4 weeks of MI training is not sufficient to improve balance indexes in older adults with high fall risk. Further research on the effectiveness of MI training and tDCS alone on balance indexes in middle-aged women is warranted. Improvements in study design could include an investigation in the MI and tDCS alone with larger sample size and longer intervention program.

## Figures and Tables

**Table 1:** Demographic variables of groups.

Variable	Intervention group (mean ± SD)	Control group (mean ± SD)	Df	*t*	*p*
Age (year)	52.46 ± 6.00	57.61 ± 5.86	28	1.10	.28
Height (cm)	160.00 ± 6.84	161.00 ± 5.41	28	1.12	.27
Weight (kg)	72.00 ± 7.95	74.00 ± 11.46	28	0.51	.61

**Table 2:** Descriptive statistics of variables.

Variable	Intervention group (mean ± SD)		Control group (mean ± SD)	
	Pretest	Posttest	Pretest	Posttest
BESS test (score)	15.92 ± 4.40	10.69 ± 2.98	16.67 ± 4.67	14.38 ± 3.25
Y test (cm)	38.69 ± 6.15	43.38 ± 5.36	36.38 ± 4.78	38.37 ± 4.71

**Table 3:** The result of ANCOVA test.

Variable	Posttest (adjusted mean ± SD)	Df	*f*	*p*	Partial *η*^2^
BESS test (score)			8.46	.01	.27
Intervention	10.79 ± 0.86	28			
Sham control	14.31 ± 0.86	28			
Y test (cm)			6.03	.02	.16
Intervention	43.42 ± 1.42	28			
Sham control	38.34 ± 1.41				

**Table 4:** The result of paired *t*-test.

Variable	Pretest	Posttest	Mean difference	Df	*t*	*p*	95% confidence interval	
							Lower	Upper
BESS test (score)				28	3.88	0.002	2.29	8.16
Intervention	15.92 ± 4.40	10.69 ± 2.98	5.23					
Sham control	16.67 ± 4.67	14.38 ± 3.25	2.38		1.17	0.11	1.36	-0.59
Y test (cm)				28	2.85	0.04	-10.19	-0.81
Intervention	38.69 ± 6.15	43.38 ± 5.36	4.69					
Sham control	36.38 ± 4.78	38.37 ± 4.71	2.01		1.25	0.23	-5.48	1.48

## Data Availability

Data is available on request from the corresponding author.
